# A Test on the Potential of a Low Cost Unmanned Aerial Vehicle RTK/PPK Solution for Precision Positioning

**DOI:** 10.3390/s21113882

**Published:** 2021-06-04

**Authors:** Nicola Angelo Famiglietti, Gianpaolo Cecere, Carmine Grasso, Antonino Memmolo, Annamaria Vicari

**Affiliations:** Istituto Nazionale di Geofisica e Vulcanologia, Osservatorio Nazionale Terremoti, Sede Irpinia, 83035 Grottaminarda AV, Italy; gianpaolo.cecere@ingv.it (G.C.); carmine.grasso@ingv.it (C.G.); antonino.memmolo@ingv.it (A.M.); annamaria.vicari@ingv.it (A.V.)

**Keywords:** PPK, UAV, drone, precision positioning, RTK, GNSS, remote sensing, photogrammetry, Multi-Base GNSS

## Abstract

This paper investigated the achievable accuracy from a low-cost RTK (Real Time Kinematic)/PPK (Post Processing Kinematic) GNSS (Global Navigation Satellite Systems) system installed on board a UAV (Unmanned Aerial Vehicle), employing three different types of GNSS Bases (Alloy, RS2 and RING) working in PPK mode. To evaluate the quality of the results, a set of seven GCPs (Ground Control Points) measured by means of the NRTK (Network Real Time Kinematic) technique was used. The outcomes show a RMSE (Root Mean Square Error) of 0.0189 m for an ALLOY Base, 0.0194 m for an RS2 Base and 0.0511 m for RING Base, respectively, on the vertical value of DEMs (Digital Elevation Models) obtained by a photogrammetric process. This indicates that, when changing the Base for the PPK, the solutions are different, but they can still be considered adequate for precision positioning with UAVs, especially when GCPs could be used with some difficulty. Therefore, the integration of a RTK/PPK GNSS module on a UAV allows the reconstruction of a highly detailed and precise DEM without using GCPs and provides the possibility to carry out surveys in inaccessible areas.

## 1. Introduction

The INGV (Istituto Nazionale di Geofisica e Vulcanologia, Italy) is often called in emergency situations related to earthquakes, landslides and volcanic eruptions. In these cases, fast mapping could provide a significant contribution during the early phases of the emergency and in general when timeliness is crucial [1,2,3,4].

Traditional methods for land mapping have some critical issues. An example of this is the difficulties that emerged during the earthquake of 2016 in central Italy when mapping field faults [5,6,7]. Other authors have used the InSAR (Interferometric Synthetic Aperture Radar) technique and GPS measurements for the same aim [8], LMRS (Landslide Mapping Remote Sensing) techniques for landslide mapping [9] or Airborne [10] and SAR [11] methods for volcano investigations.

Unmanned aerial vehicle (UAV) platforms have become a low-cost alternative to the classical methods for land mapping; these devices are equipped with precision on-board instrumentation such as IMU (an Inertial Measurement Unit) and GNSS (a Global Navigation Satellite System). UAVs are well suited to small scale and research applications, with great capacity for detailed restitution of an investigated area. These aircrafts must be considered as complete systems, as they present numerous technologies and show great investigative skills [12]. Numerous examples are present in literature in different fields of applications [13,14].

Nowadays, UAVs have become a very popular remote sensing tool for Earth observations [15,16,17].

So, UAVs are very useful platforms for scanning areas from short distances while flying at low-altitudes, but need further measurements in situ by means of precision instrumentation. Typically, these measures use GCPs (Ground Control Points) obtained by GNSS systems working in NRTK (Network Real Time Kinematic) mode, and they are successively used in the photogrammetric processing phase [15].

The accuracy of a survey made through an UAV depends on many factors, and in particular on the resolution of the sensor and the goodness of the GCPs measurements. Usually, to collect GCPs, extensive efforts are requested in terms of people and time, while also keeping in mind that areas are often inaccessible.

However, in recent years, new methodologies and techniques have been investigated and applied in this sector [16,18,19]. One of these consists of an additional precision GNSS device (the default receiver of the aircraft is used for navigation), with the aim of performing “precision geotagging” of the images.

These miniaturized devices are real Multi-Band and Multi-Constellation GNSS receivers. They provide the possibility to work with direct corrections using Real Time Kinematics or a Post Processing Kinematics mode, obtaining a high accuracy to the nearest several centimeters [20]. In the last case, it is important to know that the obtained solution is highly dependent from the GNSS base that is used for the correction.

A Base is a station where a GNSS receiver is positioned at a known location, which is also called a Reference Station or CORS (Continuously Operating Reference Station) when it is a part of a permanent GNSS net.

Therefore, this paper focused on how the use of different bases for PPK influences the precision mapping performed by means of a UAV.

## 2. Materials and Methods

In the last decade, the continuous development in the field of UAVs has made it possible to implement sophisticated and precise on-board sensors, making this type of platform more and more similar to a precision remote sensing system [21].

The main methods typically used to georeference image from a remote sensing platform are:

- IG (Indirect Georeferencing), through the use of GCP in the photogrammetric processing phase [22];
- GD (Direct Georeferencing), that takes place directly on board the vehicle using dedicated instrumentation [23].

The main tool for improving the accuracy of UAV surveys is the GNSS receiver which is mounted on board, and which can work in RTK or PPK mode.

**Figure 1:**
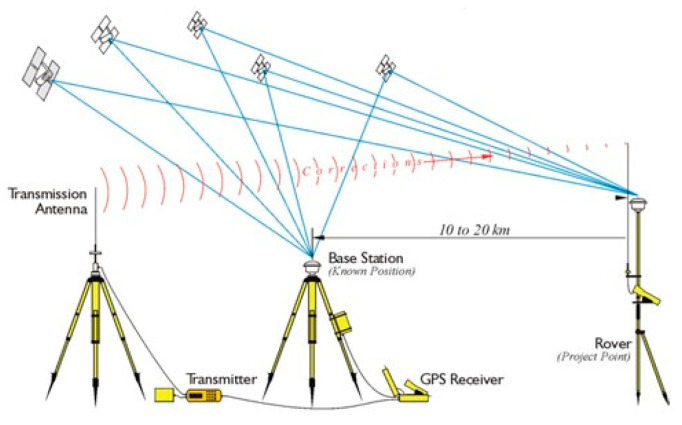
RTK system.

In the GNSS RTK data correction mode, two receivers, as shown in Figure 1, (sometimes even more than 2 in a Multi-Base configuration) are used with different functions: one is used in motion and is called Rover, while the other, called Base, has precisely the function of providing a stable support to provide positioning with excellent precision.

**Figure 2:**
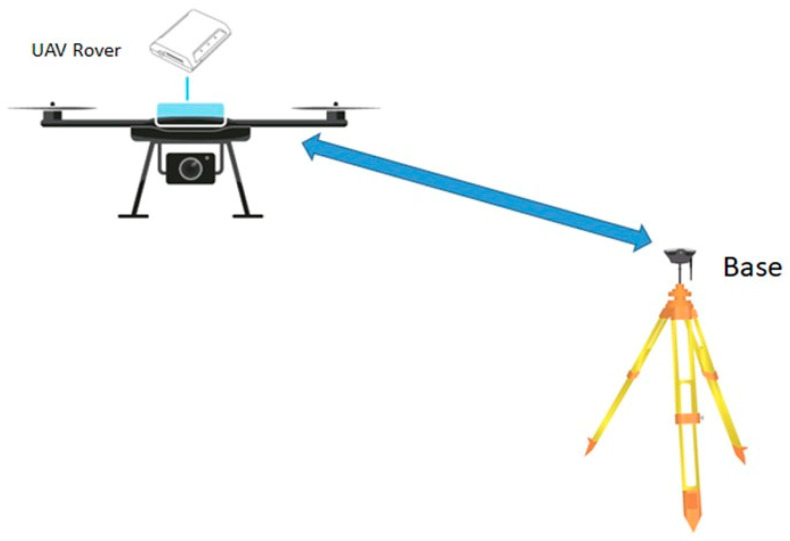
UAV Rover GNSS for RTK.

Using single frequency receivers, it is difficult to completely eliminate the effects due to the atmosphere, while with the advent of Multi-frequency receivers, this problem has been greatly reduced, even while using longer baselines (for example greater than 20 km). The possible implementation of an RTK system on a UAV is shown in Figure 2, where in this case, the rover is mounted on board a UAV and the GNSS Base is usually placed in close proximity to the flight area.

However, this RTK UAV methodology does not provide acceptable results in terms of accuracy without the use of GCPs.

**Figure 3:**
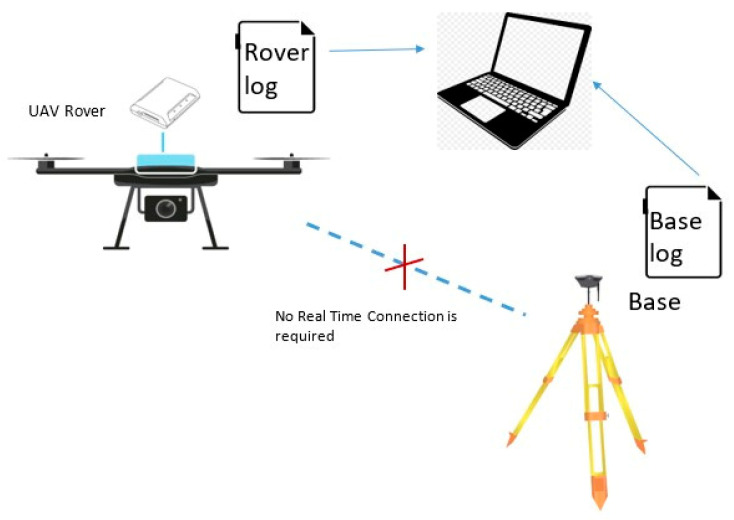
UAV PPK system.

This is a very important factor to consider when you investigate places that are not easily accessible. In this case, it is possible to exploit all the potential of the PPK, which allows acquiring the raw GNSS data of the Rover on UAV (Rover log) and of a base (Base log), and processing them comfortably in a second phase. The possible configuration of this acquisition mode is shown in Figure 3.

PPK proved to be a more flexible solution than RTK. With the PPK, it is possible to perform different types of processing of the same data several times with the aim of reducing the errors.

Furthermore, since the PPK processing does not require GCPs (Ground Control Points), it is suitable for quite large areas and also shows different changes in topographical altitude. The mapping using PPK algorithms had the same accuracy as the method involving the use of GCPs [24].

For the test case, we implemented a system that consists of a quadcopter with the following configuration:

- 4 DJI self-ventilated 310 rpm and 700 W motors;
- ESC (Electronic Speed Controller) DJI from 40 A to 26 V max (operating frequency of 30 Hz–450 Hz);
- 17 × 6.0 inch closable propellers;
- Flight Control Board DJI N3 model.

**Figure 4:**
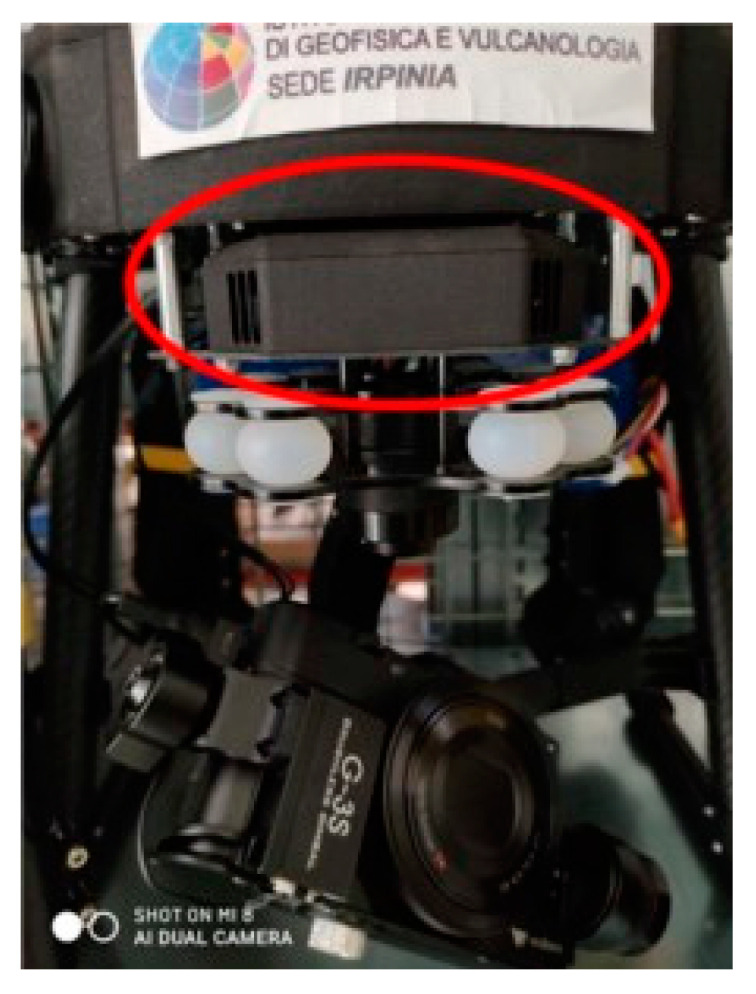
GNSS module integration on a drone.

The take-off drone weight is 6 kg and has an autonomy of about 27 min. The UAV includes a 3-axis Gimbal Brushless with an approximate weight of 500 g as support for the camera. The UAV is equipped with a Camera Sony RX100 m2, based on 1” Exmor CMOS sensor (13.2 mm × 8.8 mm) and ZEISS^®^ Vario-Sonnar T* F1.8 lens with 3.6 × optical zoom. The platform is equipped with a second compact single-GNSS receiver (Reach M+ that was used in PPK mode) as highlighted in Figure 4, with the aim to log the raw data in the UBX format using GPS and GLONASS satellites [25,26].

**Figure 5:**
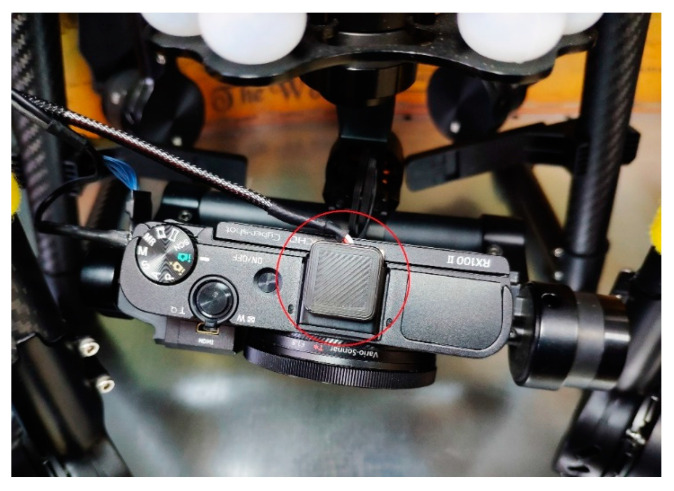
Hot-Shoe connection.

We used a “Tallysman TW4721” antenna, covering GPS/QZSS L1, GLONASS G1, Galileo E1, BeiDou B1 as well as SBAS (WAAS/EGNOS/MSAS) [27,28]. The default GNSS instrumentation mounted on a UAV was inserted with the aim of helping the navigation of the aircraft and does not have the ability to provide support for the georeferencing of images. This problem was bypassed by connecting the camera and the GNSS receiver, as shown in Figure 5, so that with each click of the camera a trigger is sent to the receiver, which records the time and coordinates with a maximum delay of one microsecond.

**Figure 6:**
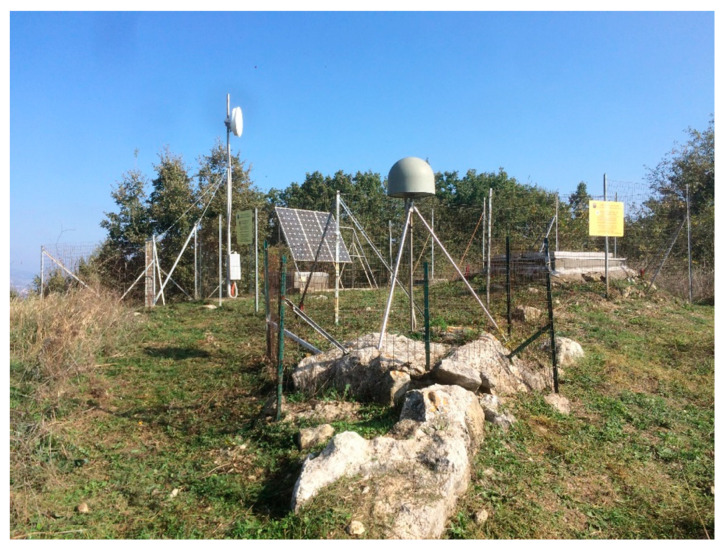
RING-PSB1 station.

The first base chosen for the test case was a site in the RING network (Integrated National GNSS Network) of INGV called **PSB1** (41°13′24.20″ N, 14°48′38.78″ E), located in the territory of Pesco Sannita (BN), as reported in Figure 6 [29].

This site of the INGV network has the following geodetic characteristics:

- Receiver: LEICA GRX1200PRO;
- Firmware: 7.50;
- Antenna: LEIAT504;
- Radome: SCIT.

The flight mission was planned using UgCS (Universal ground Control Station) software in the PRO version.

UgCS allows you to create missions directly from a kml file used as a terrain reference model and to plan them in Terrain-Following mode, thereby allowing the drone to keep its height from the ground constant throughout the flight mission [28].

**Figure 7:**
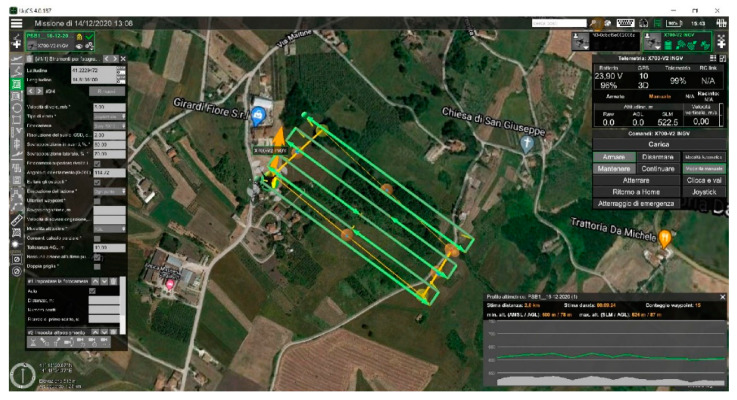
Flight mission planning.

A photogrammetric mission was planned with these parameters: flight speed 5 m/s, GSD 2 cm, Overlap 80% and Sidelap 70%, as shown in Figure 7.

The mission envisaged a flight time of 09.24 min to cover a scan area of 5.88 min. The size of the shooting area was 109.44 m × 72.96 m. A total of 15 waypoints were set and 6 passes were made. In addition, 53 shots were taken. The elevation profile showed a minimum height of 600 m/78 m (ASML/AGL) and a maximum height of 624 m/87 m (ASML/AGL), as shown in Figure 7.

On 16 December 2020, an on-site mission was organized to carry out the flight. At first, two other GNSS Bases with different receivers and antennas were installed on the site in order to carry out various forms of post-processing with different parameters.

Specifically, two configurations were put into operation:

- The first featured a Trimble Alloy professional multiband and multi-constellation receiver and a Trimble GNSS Ti-V2 Choke Ring antenna.
- The second configuration envisaged an all-in-one receiver solution of the Emlid Reach RS2 model, again with multi-band and multi-constellation characteristics, but with an integrated antenna.

**Figure 8:**
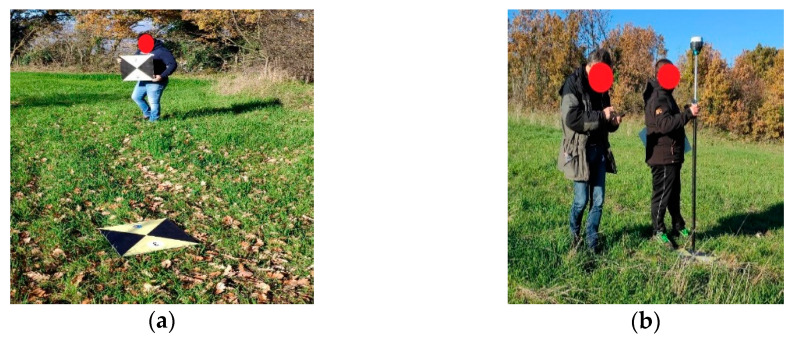
(**a**) GCPs distribution; (**b**) GCPs measurement.

The exact position of both bases was acquired with NTRIP correction and they were set with static acquisition at 5 Hz necessary for the precision post-processing of the Rover GNSS (Emlid Reach M +) data on board the drone, which was also configured with multi-frequency and multi-constellation 5 Hz RTK acquisition. After the implementation phase of the correction bases, we moved on to the distribution phase in the survey area of the GCPs and their measurement by acquiring the position coordinates with NRTK correction on the NTRIP server (see Figure 8). 

The coordinates of the bases were calculated in the reference system RTRF2000-RDN with PPK processing using the RTCM3 data of the same base (ROBS site of the Leica SmartNet network with a baseline of 20 km).

The positions of the CGPs were calculated using the IMAX3_RDN protocol of the Leica SmartNet network with RTCM3 data from the ROBS station.

In this phase, the setting of the drone, the remote control, the tablet and the notebook took place. By activating a Wi-Fi hot-spot on the tablet connected to the remote control and connecting the PC to its Wi-Fi network, all the elements were linked. 

The drone, previously configured with the GNSS receiver on board, was connected, and was thus able to receive the configuration for the scheduled flight mission. Once the drone was placed on the landing pad, a manual reconnaissance flight was carried out to check the functionality and initialize the GNSS receiver on board. Having successfully verified all the functions of the vehicle, we finally proceeded to fly with photogrammetric scanning.

**Figure 9:**
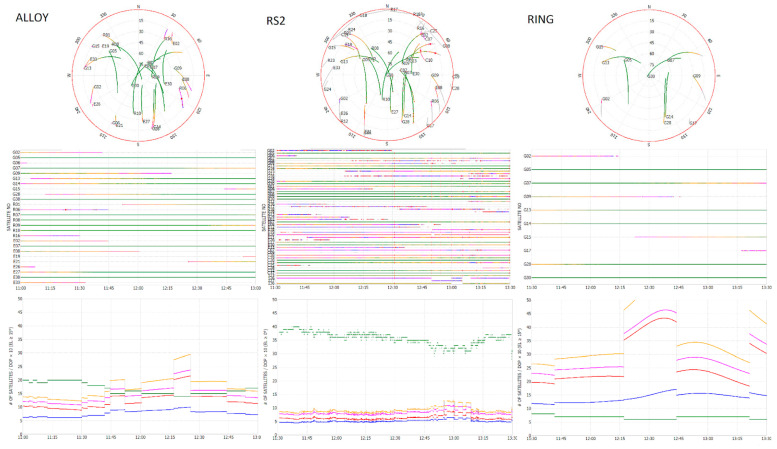
QC analysis of the observation files (timespan 11:30–13:30): L1/Lc Skyplot (top row), visibility chart (middle row) and Dilution of Precision (bottom row) of (**Left Column**) ALLOY, (**Center Column**) RS2 and (**Right Column**) RING.

The goodness of the GNSS data collected was verified, both as positions and as raw data and for both the Rover and the Bases. In particular, the RTKLIB [24,30] software was used for the QC (Quality Check) of the three different bases. For each acquisition, a satellite visibility analysis was carried out with the creation of Skyplot, Visibility Chart and SA/DOP (Satellite Availability/Dilution Of Precision). Figure 9 shows the charts of the L1/Lc Skyplot, visibility and Satellite Availability/Dilution of Precision for all 3 bases (RS2, ALLOY and RING).

It appears evident that the low DOP values of the Emlid RS2 Base are due to the large availability of satellites in sight. In fact, the average of the GDOP (Geometric Dilution Of Precision), which is roughly interpreted as the ratio between the position error and range error, is less than unity for RS2 (a), while the equivalent for Alloy (b) and RING (c) is greater than 2.

Afterwards, we entered the crucial phase of the processing: through the REDtoolbox software, we proceeded to use PPK and the GNSS data of the various Bases with the Rover GNSS on the drone and to associate the relative positions to the photos taken, producing a dedicated project (.Psx) for the Agisoft Metashape photogrammetry software [25,26].

**Figure 10:**
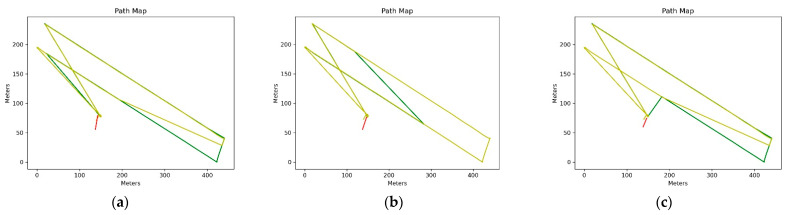
(**a**) Drone route maps with Alloy PPK correction; (**b**) Drone route maps with RS2 PPK correction; (**c**) Drone route maps with RING PPK correction.

**Figure 11:**
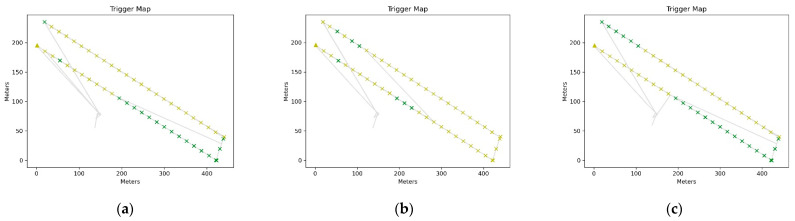
(**a**) Trigger/Drone route maps with Alloy PPK correction; (**b**) Trigger/Drone route maps with RS2 PPK correction; (**c**) Trigger/Drone route maps with RING PPK correction.

**Figure 12:**
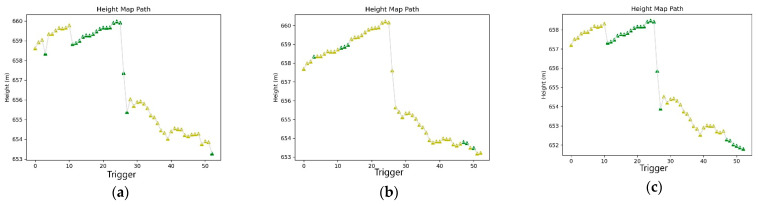
(**a**) Heights/Trigger drone maps with Alloy PPK correction; (**b**) Heights/Trigger drone maps with RS2 PPK correction; (**c**) Heights/Trigger drone maps with RING PPK correction.

At the end of this step, the complete reports of each processing were obtained containing three color-coded maps. The color scheme has the following range: green-yellow-red, where green indicates FIX solutions, yellow indicates FLOAT solutions and red indicates any other solution (see Figure 10, Figure 11 and Figure 12).

The Figure 10 contains a color-coded map of the rover’s path. It shows the path taken by the drone and the relative solution according to the color scheme indicated above.

Figure 11 contains a color-coded map of the triggers and their locations relative to the rover’s path, with each trigger point color-coded according to the previous classification (the “first” trigger is indicated with a triangle).

Figure 12 contains a diagram showing the height of each trigger, with each point again color-coded according to the previous classification.

Table 1 and Table 2 describe the number of triggers and the relative percentage for each processing of the 3 different Bases when affected by the GNSS solution quality (Fix, Float, Single, Sbas and DGPS).

**Figure 13:**
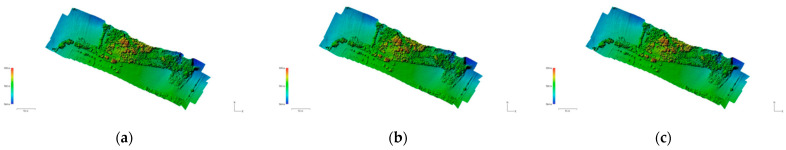
(**a**) DEM processed with Alloy PPK correction; (**b**) DEM processed with RS2 PPK correction; (**c**) DEM processed with RING PPK correction.

Once the checks of the data processed in PPK were completed, the photogrammetric processing was carried out with the production of Dense Clouds of points and DEM as shown in Figure 13.

## 3. Results

The accuracy of the results obtained was verified by analyzing the various DEMs produced in the GIS environment, then comparing them with the precision measurements made on the GCPs.

**Figure 14:**
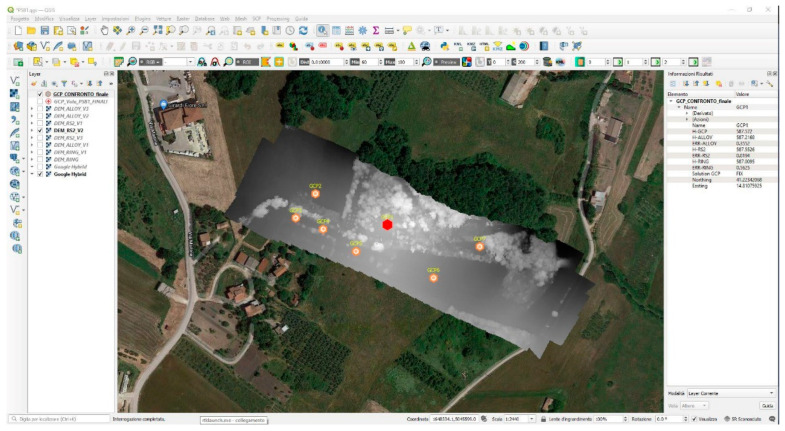
Comparison of DEM/GCPs in GIS.

In the central tab of the picture, there is a visual representation of the products with a Google basemap. In the side tabs of the picture, on the other hand, we found the list of layers on the left and information on the selected point on the right, as depicted in Figure 14. Naturally, all the layers were georeferenced in the same reference system.

From the comparison of the altimetric measurements of the various DEM obtained and the measured GCPs, the situation summarized in the following Table 3 was obtained.

Where is it:

- **NAME**, label of the measured GCP;
- **H GCP**, elevation (mt) of the GCP;
- **H ALLOY**, elevation (mt) deriving from the DEM-ALLOY in the position of the GCP;
- **E GCP ALLOY**, error deriving from the comparison of the two GCP/ALLOY quotas;
- **H RS2**, elevation (mt) deriving from DEM-RS2 in the position of the GCP;
- **E GCP RS2**, error deriving from the comparison of the two GCP/RS2 quotas;
- **H RING**, elevation (mt) deriving from the DEM-RING in the position of the GCP;
- **E GCP RING**, error deriving from the comparison of the two GCP/RING quotas;
- **S GCP, GNSS** solution of the related GCP;
- **LAT**, latitude of the GCP;
- **LON**, longitude of the GCP.

Table 3 indicates the heights (column 2) relating to the measurement of the 7 GCPs (column 1), the heights relating to the DEMs processed with the 3 different GNSS bases (columns 3, 5, 7) in the same GCPs coordinates. Columns 4, 6 and 8 show the height differences between the GCPs and the 3 Bases. Column 9 presents the solutions in NRTK mode of the GCPS measurements.

In particular, it is evident that the errors deriving from the comparison of the measured quotas of the 7 GCPs and those relating to the DEM derived from the PPK of the various bases are between 1 and 35 cm for the ALLOY, between 2 and 30 cm for the RS2 and between 5 and 56 cm for the RING base.

The comparison described in the table is also visible in the graph in the figure below.

**Figure 15:**
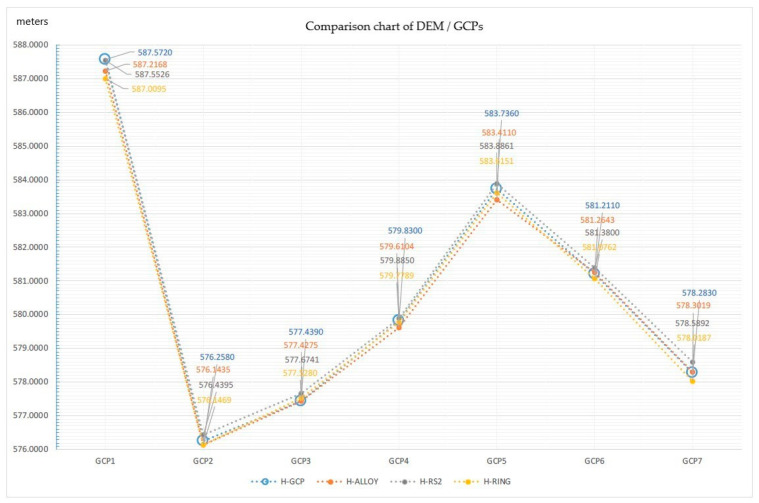
Comparison chart of DEM/GCPs.

As shown in Figure 15, the altimetric trends of the three PPK elaborations (H-ALLOY, H-RS2, H-RING) were perfectly comparable with the ones produced by RTK analyses of GCPs (H-GCP) and show reduced and acceptable deviations [27,28,29].

The graph shows that in the points GCP1 and GCP7, there were the greatest differences in terms of deviation from the measurements carried out, which however were comparable with each other. The GCp3, despite having had a FLOAT solution from the RTK measurement, did not differ much from the heights in the same points of the 3 DEMs; in fact, it remained in the same deviation range.

## 4. Discussion

This study was based on a different analysis of the results compared to the traditional approach in the photogrammetric field, where the point cloud and all the resulting products are processed by georeferencing everything through the use of GCPs.

In this work, having used more than one base for PPK, we wanted to make a comparison between the heights of the DEMs derived from the photogrammetric processing of the survey with 3 different bases for the PPK correction of the GNSS data and heights resulting from RTK mode measurements of 7 GCPs.

The used configuration of the GNSS bases were:

1. A top-of-the-range receiver for professional use, called **ALLOY**, Trimble Alloy model, Multi-frequency and Multi-constellation, connected to a professional and high-performance antenna of the Trimble GNSS Ti-V2 Choke Ring model;
2. A low cost All-in-One receiver for professional use, called **RS2**, model Emlid Reach RS2, Multi-frequency and Multi-constellation, with an internal antenna;
3. A receiver of the Italian National GNSS Network of the National Institute of Geophysics and Volcanology, called **RING**, model Leica GRX1200PRO Dual-frequency (L1-L2) and single-constellation (GPS), connected to a professional and high-performance antenna of the model Leica LEIAT504.

This approach also derives from the fact that in numerous photogrammetric investigations reconstructions carried out previously noted that a large number of GCPs well distributed over the entire ROI are required for georeferencing [31,32,33,34,35]. In fact, when soils with large differences in height are investigated, if an adequate number of GCPS is not used, then height jumps are incurred.

This is one of the factors that substantially influences a UAV precision survey, but it is not the only one. Conditions necessary for successful flight and scanning are for example environmental conditions such as temperature, sun exposure, humidity, etc. Indispensable parameters for GNSS data can for example enable an excellent coverage of the satellites, the use of several constellations and the setting of the instrumentation. In fact, before being able to carry out a test like this one, programming of all the steps well is essential.

Precisely for the reasons just mentioned, the final result is strongly influenced by the setting and programming of all the work, and therefore the results obtained must always be investigated with the maximum attention.

Furthermore, an attempt was made to adopt the same standard, with the same parameters, both for the setting of the GNSS instrumentation and for the accuracy of the positions of the Bases.

Only using the same parameters, such as for example the same GNSS Base, ROBS site of the Leica SmartNet network, RTK positioning of the GCPs and the same frequency at 5 Hz for the correct position of the bases for the PPK, etc., make it possible to make a serious comparison of the results.

The comparison of the heights deriving from the NRTK measurement of GCPS and the measurements in the same points of the DEM obtained from the PPK values of the 3 different Bases, highlighted the following results:

1. The height differences between the GCPs and the **DEM ALLOY** oscillate between the minimum of about 1 cm (for the GCP number 3) and the maximum of about 35 cm (for the GCP number 1);
2. The height differences between the GCPs and the **DEM RS2** oscillate between the minimum of about 2 cm (for GCP number 1) and the maximum of about 30 cm (for GCP number 7);
3. The height differences between the GCPs and the **DEM RING** oscillate between the minimum of about 5 cm (for the GCP number 3) and the maximum of about 56 cm (for the GCP number 1).

## 5. Conclusions

The present study aimed to investigate the potential of a GNSS receiver installed on board a drone (Emlid Reach M+) for the precision georeferencing of photogrammetric products processed without GCP with Post-Processing-Kinematics techniques with raw GNSS data derived from 3 different GNSS receivers used as Bases.

In this paper, we wanted to show that as the GNSS base for the PPK varies, the solutions also vary, but remain comparable with limited error ranges. The error was quantified by comparing the GCP measurements obtained by the NRTK technique and the various DEMs that resulted from the 3 PPK elaborations of the GNSS bases. So our goal was to investigate how the use of multiple GNSS bases affects the processing solution. It was shown that, by working in a differential way, that is with respect to a reference, different results were obtained in terms of absolute precision, but that were still perfectly comparable. The variation of outcomes between 1 and 56 cm could however be considered adequate regarding the precision positioning with UAV, especially in places where access is difficult.

The results obtained could be defined as satisfactory with good photogrammetric resolutions and acceptable positioning errors; in fact, the post processing carried out with receivers with all the constellations enabled was very stable and did not differ much from real time positioning.

Based on these results, therefore it can be said that precision mapping with a drone equipped with an on-board GNSS module does not differ much from a technique that involves the ground measurement of the GCP; indeed, it was found to be comparable in terms of errors, even in the field of heights.

The continuous technological developments in the Drones and Geodesy sectors always pushes us to search for new solutions that allow us to reduce costs, time and use of human resources, and above all, to investigate areas that are difficult to explore. In the context of rapid mapping, where timeliness is a determinant factor, the ability to completely skip the measurement of the GCPs is a clear advantage.

## Figures and Tables

**Table 1:** Percent Corrections Flight Map.

Type	ALLOY		RS2		RING	
	Total	Percentage	Total	Percentage	Total	Percentage
Fix	880	37.2%	574	24.2%	911	38.5%
Float	1485	62.7%	1792	75.7%	1454	61.4%
Single	2	0.1%	2	0.1%	2	0.1%
Sbas	0	0.0%	0	0.0%	0	0.0%
Dgps	0	0.0%	0	0.0%	0	0.0%

**Table 2:** Percentages for Corrections Flight Triggers.

Type	ALLOY		RS2		RING	
	Total	Percentage	Total	Percentage	Total	Percentage
Fix	19	36.5%	7	13.5%	23	44.2%
Float	33	63.5%	45	86.5%	29	55.8%
Single	0	0.0%	0	0.0%	0	0.0%
Sbas	0	0.0%	0	0.0%	0	0.0%
Dgps	0	0.0%	0	0.0%	0	0.0%

**Table 3:** GCP/DEM comparison.

NAME	H GCP (mt)	H ALLOY (mt)	E GCP ALLOY (mt)	H RS2 (mt)	E GCP RS2 (mt)	H RING (mt)	E GCP RING (mt)	S GCP	LAT	LON
**GCP1**	587.5720	587.2168	0.3552	587.5526	0.0194	587.0095	0.5625	Fix	41.22342068	14.81075925
**GCP2**	576.2580	576.1435	0.1145	576.4395	−0.1814	576.1469	0.1111	Fix	41.22375641	14.80972046
**GCP3**	577.4390	577.4275	0.1150	577.6741	−0.2351	577.5280	−0.0890	Float	41.22349218	14.80943705
**GCP4**	579.8300	579.6104	0.2196	579.8850	−0.0550	579.7789	0.0511	Fix	41.23337290	14.80983308
**GCP5**	583.7360	583.4110	0.3250	583.8861	−0.1501	583.6151	0.1209	Fix	41.22331391	14.81030760
**GCP6**	581.2110	581.2643	−0.0533	581.3800	−0.1690	581.0762	0.1348	Fix	41.22284603	14.81142498
**GCP7**	578.2830	578.3019	−0.0189	578.5892	−0.3062	578.0187	0.2643	Fix	41.22318432	14.81209091

## Data Availability

Not applicable.

## References

[1] Hong J.-H., Tsai C.-Y. Using 3D Webgis to support the disaster simulation, management and analysis-examples of tsunami and flood. Proceedings of the 13th GeoInformation for Disaster Management Conference.

[2] Qin R., Tian J., Reinartz P. (2016). 3D change detection-Approaches and applications. ISPRS J. Photogramm. Remote Sens..

[3] Ajmar A., Boccardo P., Disabato F., Tonolo F.G. (2015). Rapid Mapping: Geomatics role and research opportunities. Rend. Lincei.

[4] Tampubolon W., Reinhardt W. UAV data processing for rapid mapping activities. Proceedings of the ISPRS Geospatial Week 2015.

[5] Civico R., Pucci S., Villani F., Pizzimenti L., De Martini P.M., Nappi R., the Open EMERGEO Working Group (2018). Surface ruptures following the 30 October 2016 Mw 6.5 Norcia earthquake, central Italy. J. Maps.

[6] Livio F.A., Michetti A.M., Vittori E., Gregory L., Wedmore L., Piccardi L., Tondi E., Roberts G.P., Blumetti A.M., Bonadeo L. (2016). Surface faulting during the August 24, 2016, Central Italy earthquake (Mw 6.0): Preliminary results. Ann. Geophys..

[7] Gori S., Falcucci E., Galadini F., Zimmaro P., Pizzi A., Kayen R.E., Lingwall B.N., Moro M., Saroli M., Fubelli G. (2018). Surface Faulting Caused by the 2016 Central Italy Seismic Sequence: Field Mapping and LiDAR/UAV Imaging. Earthq. Spectra.

[8] Cheloni D., De Novellis V., Albano M., Antonioli A., Anzidei M., Atzori S., Avallone A., Bignami C., Bonano M., Calcaterra S. (2017). Geodetic model of the 2016 Central Italy earthquake sequence inferred from InSAR and GPS data. Geophys. Res. Lett..

[9] Zhong C., Liu Y., Gao P., Chen W., Li H., Hou Y., Nuremanguli T., Ma H. (2019). Landslide mapping with remote sensing: Challenges and opportunities. Int. J. Remote Sens..

[10] Kereszturi G., Schaefer L.N., Schleiffarth W.K., Procter J., Pullanagari R.R., Mead S., Kennedy B. (2018). Integrating airborne hyperspectral imagery and LiDAR for volcano mapping and monitoring through image classification. Int. J. Appl. Earth Obs. Geoinf..

[11] Carn S.A. (1999). Application of synthetic aperture radar (SAR) imagery to volcano mapping in the humid tropics: A case study in East Java, Indonesia. Bull. Volcanol..

[12] Nebiker S., Eugster H. (2008). UAV-based augmented monitoring-real time georeferencing and integration of video imagery with virtual globes. Int. Arch. Photogramm. Remote Sens. Spat. Inf. Sci..

[13] Remondino F., Barazzetti L., Nex F., Scaioni M., Sarazzi D. UAV photogrammetry for mapping and 3D modeling-current status and future perspectives. Proceedings of the International Archives of the Photogrammetry, Remote Sensing and Spatial Information Sciences.

[14] Kerle N., Nex F., Gerke M., Duarte D., Vetrivel A. (2020). UAV-Based Structural Damage Mapping: A Review. ISPRS Int. J. Geo-Inf..

[15] Rosnell T., Honkavaara E. (2012). Point Cloud Generation from Aerial Image Data Acquired by a Quadrocopter Type Micro Unmanned Aerial Vehicle and a Digital Still Camera. Sensors.

[16] Gabrlik P. (2015). The Use of Direct Georeferencing in Aerial Photogrammetry with Micro UAV. IFAC-PapersOnLine.

[17] Tarolli P. (2014). High-resolution topography for understanding Earth surface processes: Opportunities and challenges. Geomorphology.

[18] Stempfhuber W., Buchholz M. A precise, low-cost rtk gnss system for uav applications. Proceedings of the International Archives of the Photogrammetry, Remote Sensing and Spatial Information Sciences.

[19] Turner D., Lucieer A., Watson C. (2012). An Automated Technique for Generating Georectified Mosaics from Ultra-High Resolution Unmanned Aerial Vehicle (UAV) Imagery, Based on Structure from Motion (SfM) Point Clouds. Remote Sens..

[20] Isejima J., Takasu T., Ebinuma T., Yasuda A. (2008). Performance Evaluation of the RTK-GPS Positioning with Communication Delay. J. Jpn. Inst. Navig..

[21] Eisenbeiß H. (2009). UAV Photogrammetry. Ph.D. Thesis.

[22] D’Oleire-Oltmanns S., Marzolff I., Peter K.D., Ries J.B. (2012). Unmanned Aerial Vehicle (UAV) for Monitoring Soil Erosion in Morocco. Remote Sens..

[23] Passalacqua P., Belmont P., Staley D.M., Simley J.D., Arrowsmith J.R., Bode C.A., Crosby C., DeLong S.B., Glenn N.F., Kelly S.A. (2015). Analyzing high resolution topography for advancing the understanding of mass and energy transfer through landscapes: A review. Earth-Sci. Rev..

[24] Zhang H., Aldana-Jague E., Clapuyt F., Wilken F., Vanacker V., Van Oost K. (2019). Evaluating the Potential of PPK Direct Georeferencing for UAV-SfM Photogrammetry and Precise Topographic Mapping. Earth Surf. Dynam. Discuss..

[25] Nagai M., Chen T., Shibasaki R., Kumagai H., Ahmed A. (2009). UAV-Borne 3-D Mapping System by Multisensor Integration. IEEE Trans. Geosci. Remote Sens..

[26] Dinkov D., Kitev A. Advantages, Disadvantages and Applicability of Gnss Post-Processing Kinematic (Ppk) Method for Direct Georeferencing of Uav Images. Proceedings of the 8th International Conference on Cartography and GIS.

[27] Valente D.S.M., Momin A., Grift T., Hansen A. (2020). Accuracy and precision evaluation of two low-cost RTK global navigation satellite systems. Comput. Electron. Agric..

[28] Takasu T., Yasuda A. Evaluation of RTK-GPS Performance with Low-cost Single-frequency GPS Receivers. Proceedings of the International Symposium on GPS/GNSS.

[29] Avallone A., Selvaggi G., D’Anastasio E., D’Agostino N., Pietrantonio G., Riguzzi F., Serpelloni E., Anzidei M., Casula G., Cecere G. (2010). The RING network: Improvement of a GPS velocity field in the central Mediterranean.INGV, Istituto Nazionale di Geofisica e Vulcanologia. Ann. Geophys..

[30] Takasu T., Kubo N., Yasuda A. (2007). Development, evaluation and application of RTKLIB: A program library for RTK-GPS. GPS/GNSS Symp..

[31] Roegner G.C., Coleman A.M., Borde A.B., Tagestad J.D., Erdt R., Aga J., Zimmerman S.A., Cole C. (2019). Quantifying Restoration of Juvenile Salmon Habitat with Hyperspectral Imaging from an Unmanned Aircraft System of Report.

[32] Tomaštík J., Mokroš M., Saloň Š., Chudý F., Tunák D. (2017). Accuracy of Photogrammetric UAV-Based Point Clouds under Conditions of Partially-Open Forest Canopy. Forests.

[33] Taddia Y., González-García L., Zambello E., Pellegrinelli A. (2020). Quality Assessment of Photogrammetric Models forFaçade and Building Reconstruction Using DJI Phantom 4 RTK. Remote Sens..

[34] Tomaštík J., Mokroš M., Surový P., Grznárová A., Merganič J. (2019). UAV RTK/PPK Method—An Optimal Solution for Mapping Inaccessible Forested Areas?. Remote Sens..

[35] Turner D., Lucieer A., Wallace L. (2014). Direct georeferencing of ultrahigh-resolution UAV imagery. IEEE Trans. Geosci. Remote Sens..

